# Comparative Genomics Suggests that the Fungal Pathogen *Pneumocystis* Is an Obligate Parasite Scavenging Amino Acids from Its Host's Lungs

**DOI:** 10.1371/journal.pone.0015152

**Published:** 2010-12-20

**Authors:** Philippe M. Hauser, Frédéric X. Burdet, Ousmane H. Cissé, Laurent Keller, Patrick Taffé, Dominique Sanglard, Marco Pagni

**Affiliations:** 1 Institute of Microbiology, Centre Hospitalier Universitaire Vaudois and University of Lausanne, Lausanne, Switzerland; 2 Vital-IT Group, Swiss Institute of Bioinformatics, Lausanne, Switzerland; 3 Département d'Écologie et Évolution, University of Lausanne, Lausanne, Switzerland; 4 Data Center, Swiss HIV Cohort Study, Centre Hospitalier Universitaire Vaudois and University of Lausanne, Lausanne, Switzerland; University of California Riverside, United States of America

## Abstract

*Pneumocystis jirovecii* is a fungus causing severe pneumonia in immuno-compromised patients. Progress in understanding its pathogenicity and epidemiology has been hampered by the lack of a long-term *in vitro* culture method. Obligate parasitism of this pathogen has been suggested on the basis of various features but remains controversial. We analysed the 7.0 Mb draft genome sequence of the closely related species *Pneumocystis carinii* infecting rats, which is a well established experimental model of the disease. We predicted 8’085 (redundant) peptides and 14.9% of them were mapped onto the KEGG biochemical pathways. The proteome of the closely related yeast *Schizosaccharomyces pombe* was used as a control for the annotation procedure (4’974 genes, 14.1% mapped). About two thirds of the mapped peptides of each organism (65.7% and 73.2%, respectively) corresponded to crucial enzymes for the basal metabolism and standard cellular processes. However, the proportion of *P. carinii* genes relative to those of *S. pombe* was significantly smaller for the “amino acid metabolism” category of pathways than for all other categories taken together (40 versus 114 against 278 versus 427, P<0.002). Importantly, we identified in *P. carinii* only 2 enzymes specifically dedicated to the synthesis of the 20 standard amino acids. By contrast all the 54 enzymes dedicated to this synthesis reported in the KEGG atlas for *S. pombe* were detected upon reannotation of *S. pombe* proteome (2 versus 54 against 278 versus 427, P<0.0001). This finding strongly suggests that species of the genus *Pneumocystis* are scavenging amino acids from their host's lung environment. Consequently, they would have no form able to live independently from another organism, and these parasites would be obligate in addition to being opportunistic. These findings have implications for the management of patients susceptible to *P. jirovecii* infection given that the only source of infection would be other humans.

## Introduction

Fungi of the genus *Pneumocystis* each infect a unique mammalian species [1], [2]. Although *P. jirovecii* infecting humans is the most frequent AIDS-defining pneumonia and a major cause of mortality in immuno-compromised patients [3], progress in understanding its pathogenicity and epidemiology has been hampered by the lack of a long-term *in vitro* culture method. In that respect, it is crucial to know whether species of the genus *Pneumocystis* are obligate parasites depending strictly on their host, or if they have a form capable of replicating in nature independently of other organisms [4]. Obligate parasitism has been suggested on the basis of their strict host specificity [5]–[7], patterns of co-evolution with hosts [5], [8], genetic flexibility of chromosome ends responsible for expression of a single antigen encoding gene [9], [10], and the fact that they scavenge cholesterol from their host to build their own membranes [11]. Scavenged cholesterol is found in the membrane together with specific sterols that *Pneumocystis* synthesizes *de novo* [12]. However, the issue of whether *Pneumocystis* species also have a free-living form in nature remains controversial. Indeed, closely related plant pathogens of the genus *Taphrina* also show strict host specificity and co-evolution with hosts [13], yet they have free-living forms.

The loss of biosynthetic pathways of essential molecules such as amino acids, co-factors, nucleotides, and/or vitamins is a hallmark of obligate humans' parasites, such as *Encephalitozoon cuniculi* [14], *Plasmodium falciparum* [15], [16], *Cryptosporidium hominis* [15], *Leishmania major* [15], *Coxiella burnetti* [17], and *Legionella pneumophila* [18]. Unambiguous proof that a parasite does not have a free-living form can thus be obtained from the demonstration that it has lost such vital functions. The almost completed *Pneumocystis carinii* genome (http://pgp.cchmc.org), which is a very close relative of *P. jirovecii* infecting rats, provides an opportunity to investigate whether species of this genus have lost essential cellular functions making them obligate parasites. In the present study, we analysed the *P. carinii* draft genome using that of the closely related yeast *Schizosaccharomyces pombe* as a control for the annotation procedure.

## Results and Discussion

The draft genome of *P. carinii* totalizes ca. 7.0 Mb. It is made of numerous unassembled contigs and covers 70 to 100% of the whole genome on the basis of karyotype analyses. We predicted 8’085 (redundant) peptides corresponding to approximately 4’000 complete or partial protein-coding genes using a gene model designed for Augustus software [19]. The predicted protein sequences were mapped onto the KEGG biochemical pathways using blast best hits against *Yarrowia lipolytica* and *Neosartorya fischeri* NRRL 181. The selection of this pair of reference proteomes was critical to ensure the best annotation results (see Methods). The proteome of the yeast *S. pombe* was used as a control in the mapping procedure. This species is the closest relative of *Pneumocystis* species with a sequenced genome, as it is also a member of the lineage Archiascomycetes. The latter is one of the three major lineages of the Ascomycetes (archi-, hemi- and euascomycetes), and includes also free-living and plant parasitic yeasts [20].

Among the peptides we predicted, 1205 for *P. carinii* (14.9% of 8’085 peptides) and 701 for *S. pombe* (14.1% of 4’974 genes) were annotated and mapped into the KEGG atlas of biochemical pathways. About two thirds of the peptides of each organism (65.7% [792] and 73.2% [513], respectively) were mapped into 56 pathways corresponding to the basal metabolism and standard cellular processes (Table 1). In agreement with transcriptome data [21], numerous and crucial *P. carinii* enzymes were identified for the metabolism of carbohydrate, energy, lipid, nucleotide, amino acids, glycans, cofactors, and vitamins, as well as for transcription, translation, cell cycle, DNA metabolism, and various important cellular processes. However, genes identified in “amino acid metabolism” pathways were underrepresented. This category comprised 114 genes for *S. pombe* but only 40 for *P. carinii*. Accordingly, the proportion of *P. carinii* genes relatively to those of *S. pombe* was significantly smaller for this category of genes (40 versus 114 [35.1%]) than for all other categories taken together (278 versus 427 [65.1%], P<0.002, test for two binomial proportions).

**Table 1:** Number of KEGG orthologs (KO) predicted for *P. carinii* and *S. pombe* in 56 pathways that correspond to basal metabolism and cellular processes^a^.

			Number of KOs			
		Map no.	*S. pombe* (reference)	*S. pombe*	*P. carinii*	*P. carinii/S. pombe*
Carbohydrate Metabolism						
	Glycolysis/Gluconeogenesis	10	23	22	14	0.64
	Citrate cycle (TCA cycle)	20	21	20	19	0.95
	Pentose phosphate pathway	30	15	15	8	0.53
	Fructose and mannose metabolism	51	11	10	8	0.80
	Galactose metabolism	52	10	7	4	0.57
	Starch and sucrose metabolism	500	15	11	12	1.09
	Amino sugar and nucleotide sugar metabolism	520	15	14	10	0.71
	Inositol phosphate metabolism	562	7	6	6	1.00
	Pyruvate metabolism	620	19	16	12	0.75
	Glyoxylate and dicarboxylate metabolism	630	7	4	3	0.75
	Propanoate metabolism	640	9	8	4	0.50
	Butanoate metabolism	650	9	8	4	0.50
	OVERALL KOs		101	86	62	0.72
Energy Metabolism						
	Oxidative phosphorylation	190	47	41	46	1.12
	Carbon fixation in photosynthetic organisms	710	11	11	9	0.82
	Reductive carboxylate cycle (CO2 fixation)	720	6	6	4	0.67
	Nitrogen metabolism	910	9	9	3	0.33
	Sulfur metabolism	920	11	8	2	0.25
	OVERALL KOs		82	73	63	0.86
Lipid Metabolism						
	Fatty acid biosynthesis	61	6	5	4	0.80
	Steroid biosynthesis	100	13	12	9	0.75
	Glycerolipid metabolism	561	6	3	3	1.00
	Glycerophospholipid metabolism	564	16	13	7	0.54
	Ether lipid metabolism	565	5	2	1	0.50
	Sphingolipid metabolism	600	7	5	2	0.40
	Biosynthesis of unsaturated fatty acids	1040	5	4	4	1.00
	OVERALL KOs		52	41	28	0.68
Nucleotide Metabolism						
	Purine metabolism	230	59	55	37	0.67
	Pyrimidine metabolism	240	47	44	32	0.73
	OVERALL KOs		77	72	48	0.67
Amino Acid Metabolism						
	Alanine, aspartate and glutamate metabolism	250	20	19	8	0.42
	Glycine, serine and threonine metabolism	260	21	20	7	0.35
	Cysteine and methionine metabolism	270	22	18	4	0.22
	Valine, leucine and isoleucine degradation	280	5	5	3	0.60
	Valine, leucine and isoleucine biosynthesis	290	13	14	5	0.36
	Lysine biosynthesis	300	10	7	0	-
	Lysine degradation	310	11	10	7	0.70
	Arginine and proline metabolism	330	26	24	3	0.13
	Histidine metabolism	340	8	9	0	-
	Tyrosine metabolism	350	8	7	2	0.29
	Phenylalanine metabolism	360	6	6	1	0.17
	Tryptophan metabolism	380	6	6	4	0.67
	Phenylalanine, tyrosine and tryptophan biosynthesis	400	20	15	8	0.53
	OVERALL KOs		128	114	40	0.35
Metabolism of Other Amino Acids						
	beta-Alanine metabolism	410	5	5	0	-
	Selenoamino acid metabolism	450	10	8	4	0.50
	Cyanoamino acid metabolism	460	6	5	0	-
	Glutathione metabolism	480	13	13	7	0.54
	OVERALL KOs		31	28	11	0.39
Glycan Biosynthesis and Metabolism						
	N-Glycan biosynthesis	510	18	16	11	0.69
	Glycosylphosphatidylinositol(GPI)-anchor biosynthesis	563	8	8	5	0.63
	OVERALL KOs		26	24	16	0.67
Metabolism of Cofactors and Vitamins						
	Ubiquinone and other terpenoid-quinone biosynthesis	130	5	3	3	1.00
	One carbon pool by folate	670	12	9	5	0.56
	Riboflavin metabolism	740	8	6	4	0.67
	Vitamin B6 metabolism	750	7	7	4	0.57
	Nicotinate and nicotinamide metabolism	760	6	5	3	0.60
	Pantothenate and CoA biosynthesis	770	9	9	1	0.11
	Folate biosynthesis	790	7	3	2	0.67
	Porphyrin and chlorophyll metabolism	860	14	13	11	0.85
	OVERALL KOs		67	54	32	0.59
Transcription						
	RNA polymerase	3020	17	17	12	0.71
	Spliceosome	3040	12	12	11	0.92
	OVERALL KOs		29	29	23	0.79
Translation						
	Aminoacyl-tRNA biosynthesis	970	24	24	21	0.88
	GENERAL OVERALL KOs		485	427	278	0.65

a The reference gene numbers of *S. pombe* are those obtained from KEGG. Maps with less than five reference genes of *S. pombe* are not shown. KOs which are redundant in the pathways are counted only once in “OVERALL KOs”.

Importantly, a further analysis revealed that many genes responsible for the metabolism of the 20 standard amino acids were present, but all except two of those involved in their biosynthesis were lacking in *P. carinii*. Overall, we identified only two orthologues (EC 2.6.1.1, Aspartate transaminase; EC 1.4.1.2, Glutamate dehydrogenase) of the 54 genes specifically dedicated to the amino acids biosyntheses reported in KEGG for *S. pombe*. By contrast, all these 54 genes were identified upon reannotation of the *S. pombe* proteome (Table 2). The genes dedicated to these biosyntheses identified in *P. carinii* were greatly underrepresented relatively to those of *S. pombe* (2 versus 54 [3.7%] against 278 versus 427 [65.1%], P<0.0001, test for two binomial proportions). The non-detection of these genes could also not be accounted for by the clustering of their loci, as genomic data show that they are not clustered but dispersed all over the genome in the close fungi *S. pombe* (http://old.genedb.org/genedb/pombe/), *Saccharomyces cerevisiae* (http://www.yeastgenome.org/), *Aspergillus* (http://www.aspgd.org/), and *Neurospora crassa* (http://www.broadinstitute.org/annotation/genome/neurospora/MultiHome.html).

**Table 2:** Number of enzymes dedicated to the biosyntheses of amino acids identified in *P. carinii* and *S. pombe* a.

	No of dedicated enzymes		
Amino acid	in *S. pombe* (reference)	in *S. pombe*	in *P. carinii*
Ala	1	1	0
Asp	1	1	1
Asn	1	1	0
Arg	3	3	0
Cys	2	2	0
Glu	1	1	1
Gln	1	1	0
Gly	1	1	0
His	6	6	0
Ile^b^	4	4	0
Leu^c^	3	3	0
Lys from aspartate	0	0	0
Lys from pyruvate	7	7	0
Met	3	3	0
Phe	2	2	0
Pro	1	1	0
Ser	3	3	0
Thr from glycine	1	1	0
Thr from homoserine	2	2	0
Trp	5	5	0
Tyr	2	2	0
Val^b^	4	4	0
TOTAL	54	54	2

a The reference gene numbers of *S. pombe* are those obtained from KEGG.

b The four enzymes are the same for Ile and Val syntheses.

c One of the enzymes is also involved in Ile and Val syntheses.

Obligate parasitism of *P. carinii* would be consistent with its small genome size and low gene content relative to those of the closely related free-living fungi *S. pombe* and *S. cerevisiae* (Table 3). The evolution of obligate parasitism and loss of biosynthetic pathways has been shown to result in genome size reduction in both eukaryotic and prokaryotic obligate parasites [22], [23]. Compaction by reduction of intergenic space and number of introns has also been documented in *P. carinii* and *E. cuniculi*, respectively [24]. The microsporidian fungi are extreme cases of eukaryotic obligate parasitism scavenging several essential compounds from humans, i.e. amino acids, nucleotides, lipids, and vitamins [13], and yet they harbour the smallest known eukaryotic genomes, 2.3 Mb and ca. 2’000 genes for *E. intestinalis* [25]. Other eukaryotic obligates parasites depend on their host for fewer molecules and have larger genomes (Table 3). *P. falciparum*, *L. major,* and *C. hominis* scavenge amino acids [15], [16], whereas *Pneumocystis* species would scavenge at least amino acids and cholesterol [11]. The composition of the extracellular host environment, or of several hosts' environments for some parasites, probably determines the extent of gene loss. *C. hominis* and *Pneumocystis* species may have lost more genes than *P. falciparum* and *L. major*, possibly 20 to 30% of the genome of their free-living ancestor, because they have a single host rather than two. The presence of a single rRNA operon in *P. carinii* genome [26], the unique example among fungi, may constitute a specific adaptation to the lung environment.

**Table 3:** Some features of free-living microorganisms and obligate parasites.

	Genome size (approx. Mb)	Gene content (approx. no.)	Number of rDNA loci (approx. no.)	Minimum metabolic requirements	Number of hosts
*S. cerevisiae*	13	6300	150	none	0
*S. pombe*	14	5000	120	none	0
*P. falciparum*	23	5300	4–8	amino acids	2
*L. major*	33	6200	20–60	amino acids	2
*C. hominis*	10	4000	4–5	amino acids	1
*P. carinii*	8	4000	1	amino acids cholesterol	1
*E. cuniculi*	3	2000	20	amino acids nucleotides lipids vitamins	1

The multiple amino acid requirements of *P. carinii* suggested here implies that *Pneumocystis* species may have no form able to live independently from another organism, and thus that these parasites are obligate in addition to being opportunistic. *P. jirovecii* would be together with *Candida* species and the dermatophytes among the few Ascomycetes that can be described, in the present state of the knowledge, as obligate parasites. Obligate parasitism would have important implications for the management of patients susceptible to *P. jirovecii* infection because the only source of infection of this pathogen to be protected from would be humans. The proteolytic activity of *Pneumocystis* species [27], their surface proteases [28], their amino acid [29] and oligopeptide (our unpublished observation) permeases, may be involved in scavenging amino acids, as described in other Ascomycetes [30]. These processes would constitute new virulence factors contributing to pathogenicity and which may be used as targets for pharmaceutical intervention. The effect of HIV protease inhibitors on *P. carinii* [31] may reflect inhibition of these processes. Finally, understanding *Pneumocystis*' metabolic requirements may help to develop a method of *in vitro* growth of these fungi. Nevertheless, many unsuccessful attempts of growth in presence of amino acids have been reported [2], suggesting that other factors are required to promote their growth.

## Methods

### *P. carinii* gene prediction

The sequences of the draft genome of *P. carinii* were retrieved from the *Pneumocystis* genome project website (http://pgp.cchmc.org/). They consisted of 4’278 contigs totaling 6’345’403 bps and were accompanied with 1043 ESTs totaling 1’416’543 bps. These sequences are considered by M.T. Cushion (personal communication) to cover approximately 90% of the *P. carinii* genome which consists of ca. 8 Mb on the basis of karyotype analyses [32]. Complementary Illumina sequences consisting of 4’426 contigs totalling 4’408’129 bps and presenting 86% of overlap with those of the genome project were also obtained from M.T. Cushion. Altogether, the sequences analyzed here are estimated to include at least 7.0 Mb of unique sequences covering 70 to 100% of the whole *P. carinii* genome. Repetitive sequences may have been missed in these sets of sequences but they are thought to be scarce in fungi [33], [34].

Initially, 70 annotated genes of *P. carinii* were retrieved from Genbank. They have been used to train a gene model for SNAP [35], a gene-prediction program suitable for small training set. Preliminary investigations of the predicted pathways revealed that some proteins of the “standard” pathways (e.g., the TCA cycle) were actually missed by SNAP. A few of these missed genes were manually annotated on the contigs based on the alignment of the closest fungal homologs using GeneWise [36]. The training set was completed and a better gene model was then built for SNAP. In parallel, an *ab initio* gene model was produced using GeneMark-ES Ver. 2.3 [37]. We then supplied both the SNAP and the GeneMark gene models, together with the *P. carinii* contigs and ESTs, to the MAKER pipeline for genome annotation [38]. In addition to attempting to reconcile the gene predictions from the different models, MAKER also considers the exon evidences obtained from the mapping of the ESTs, and from the UniProt protein homologies. MAKER returned the predictions of 2’566 genes on the *P. carinii* contigs. These genes were most often consistent with the predictions by SNAP. However, SNAP and MAKER can only produce prediction of complete gene (i.e. genes that are incomplete because they are located at an extremity of a contig cannot be detected, or portions of them are wrongly reported as complete). Based on the MAKER gene annotations, i.e. a much larger set of genes that was initially available, we built a gene model for Augustus [19], which is a gene-prediction program capable to annotate properly an incomplete gene located at the extremity of a contig. It should be noted that Augustus is distributed with a gene model for *S. pombe*, that we did not find working well on *Pneumocystis* contigs. This overall gene prediction strategy eventually yielded a total of 3’977 complete or partial genes from the contigs of *P. carinii*. Augustus was also used to detect the correct reading frame in the ESTs and yield an additional 1’211 coding sequences, mostly incomplete and also mostly redundant with those already predicted from the contigs. The illumina sequences yielded 2’897 peptides. The whole procedure eventually yielded 8’085 predicted peptides with an average length of 287 amino acids. We estimate that they account roughly for about four thousands distinct protein-coding genes.

### Mapping into KEGG

The *P. carinii* predicted proteome was compared to 18 complete fungal proteomes listed in Table 4 and to *Dictyostelium discoideum* proteome, using the blastp program [39] with default parameter values and a Bit-score threshold of 45. This yielded 638’304 pairwise alignments that were stored in HitKeeper [40], our relational database management system dedicated to sequence analysis. For every fungal proteome, the collection of the “KEGG Orthologs” [41] (KO) were also stored in HitKeeper, and provided the mappings between the proteins and the KEGG biochemical pathways. Given one or several “reference” proteomes as intermediary data set, the highest scoring blastp matches was retained for every *P. carinii* peptide. Reciprocal best hits were not considered because of the fragmented and partially redundant nature of the predicted *P. carinii* proteins.

**Table 4:** Proteomes investigated for transfer the KEGG annotations of the *P. carinii* predicted proteome.

*Dictyostelium discoideum*
*Schizosaccharomyces pombe*
*Encephalitozoon cuniculi*
*Ustilago maydis*
*Filobasidiella neoformans*
*Yarrowia lipolytica*
*Candida glabrata*
*Candida albicans*
*Kluyveromyces lactis*
*Pichia stipitis*
*Saccharomyces cerevisiae*
*Debaryomyces hansenii*
*Vanderwaltozyma polyspora* DSM 70294
*Eremothecium gossypii*
*Neurospora crassa*
*Magnaporthe grisea*
*Botryotinia fuckeliana* B05.10
*Aspergillus niger* CBS 513.88
*Aspergillus oryzae*
*Neosartorya fischeri* NRRL 181

**Figure 1:**
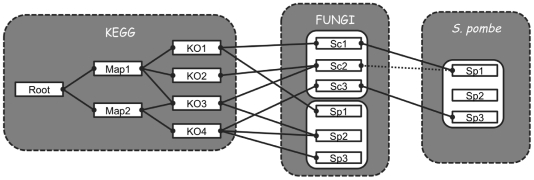
Principle of the numerical experience used to optimize the precision and recall of the annotation predictions. The *S. pombe* proteome (right box) was blasted against an intermediary set of fungal proteins, i.e. the proteome of *S. cerevisiae* in this example (middle box), and only the highest scoring blast matches were retained. By utilizing the *S. cerevisiae* mapping to the KEGG Orthologs (between the middle and left boxes), one can produce a mapping through *S. cerevisiae* of the *S. pombe* proteins to the KEGG Orthologs. The latter mapping can then be compared with the one that is actually provided by KEGG to compute precision and recall values. The experience was systematically repeated using different proteomes as intermediary data sets (or several proteomes at once), to eventually determine the optimal one.

**Figure 2:**
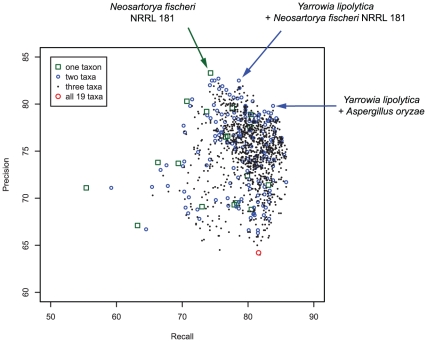
Estimation of the quality of the mapping onto KEGG maps by performing a re-prediction of the annotation of *S. pombe* proteome through intermediary data set consisting of one, two, three, or 18 fungal proteomes. The KO - *S. pombe* association pairs obtained by “blasting” an intermediary data set were evaluated *a posteriori* as true positive (TP) or false positive (FP) according to the KO - *S. pombe* mapping which is provided by KEGG. Those missed KO - *S. pombe* pairs existing in KEGG were taken as false negatives (FN). The overall quality of the obtained mapping can be expressed in terms of precision TP/(TP+FP) and recall TP/(TP+FN).

Preliminary investigations showed that the most critical parameter in this annotation procedure was the choice of the intermediary organism(s), and not the blast parameters or the score threshold, for example. Indeed, a non-negligible amount of internal inconsistencies and mapping errors are known to be present in KEGG, as well as in many other databases with the same scope [42]. One could have conjectured that an organism that is taxonomically close to *Pneumocystis* should have been chosen. However, the exhaustiveness and internal consistency of the KEGG annotations proved highly variable among the different organisms. Utilizing more than one proteome as intermediary data set is easy to implement with HitKeeper, but its benefits in term of annotation transfer cannot be easily predicted. To determine the best intermediary set to use, we attempted to re-predict the annotation of the *S. pombe* proteome, through one, two, three or all the 18 proteomes. The principle of this numerical experience is presented in Figure 1. The results of these simulations are presented in Figure 2 and reveal that the choice of the intermediary data set has a profound influence on mapping precision and recall. With a single species, the best results were obtained with *Neosartorya fischeri* NRRL 181. When two organisms were considered as forming the intermediary data sets, the best pairs turned out to be *Yarrowia lipolytica* + *N. fischeri* NRRL 181 on the one hand, and *Y. lipolytica* + *Aspergillus oryzae* on the other hand. No further improvement was observed for any possible trios of organisms. When all species were used as the intermediary data sets, a serious decrease in the precision was observed, while the coverage remained acceptable. These simulation results were obtained with data downloaded from KEGG on the 15^th^ January 2010. The strategy for selecting the optimal intermediary data set was repeated with a different release of KEGG, and yielded a distinct “optimal data reference set”. However, it led exactly the same conclusions regarding *Pneumocystis* biochemistry.

Our *Pneumocytis* prediction parameters are included in the release of Augustus software as well as on the Augustus website (http://augustus.gobics.de/). The peptides we predicted as well as their annotations are posted on P. Hauser's web page (http://www.chuv.ch/imul/imu_home/imu_recherche/imu_recherche_hauser.htm), as well as on the *Pneumocystis* genome project website (http://pgp.cchmc.org/).
